# HGF/c-MET Signaling in Melanocytes and Melanoma

**DOI:** 10.3390/ijms19123844

**Published:** 2018-12-03

**Authors:** Malgorzata Czyz

**Affiliations:** Department of Molecular Biology of Cancer, Medical University of Lodz, 6/8 Mazowiecka Street, 92-215 Lodz, Poland; malgorzata.czyz@umed.lodz.pl; Tel.: +48-42-272-57-02

**Keywords:** HGF, MET, skin, melanocytes, melanoma, drug resistance, invasive growth, wound healing, tumor microenvironment

## Abstract

Hepatocyte growth factor (HGF)/ mesenchymal-epithelial transition factor (c-MET) signaling is involved in complex cellular programs that are important for embryonic development and tissue regeneration, but its activity is also utilized by cancer cells during tumor progression. HGF and c-MET usually mediate heterotypic cell–cell interactions, such as epithelial–mesenchymal, including tumor–stroma interactions. In the skin, dermal fibroblasts are the main source of HGF. The presence of c-MET on keratinocytes is crucial for wound healing in the skin. HGF is not released by normal melanocytes, but as melanocytes express c-MET, they are receptive to HGF, which protects them from apoptosis and stimulates their proliferation and motility. Dissimilar to melanocytes, melanoma cells not only express c-MET, but also release HGF, thus activating c-MET in an autocrine manner. Stimulation of the HGF/c-MET pathways contributes to several processes that are crucial for melanoma development, such as proliferation, survival, motility, and invasiveness, including distant metastatic niche formation. HGF might be a factor in the innate and acquired resistance of melanoma to oncoprotein-targeted drugs. It is not entirely clear whether elevated serum HGF level is associated with low progression-free survival and overall survival after treatment with targeted therapies. This review focuses on the role of HGF/c-MET signaling in melanoma with some introductory information on its function in skin and melanocytes.

## 1. Introduction

HGF (hepatocyte growth factor), also known as scatter factor and tumor cytotoxic factor, is a large multidomain heterodimeric protein that belongs to the cytokine family [1]. HGF was discovered a long time ago [2], and soon after, *HGF* was cloned and expressed [3,4]. Evidence for the identity of hepatocyte growth factor, scatter factor, and tumor cytotoxic factor was provided shortly after [5,6]. The gene encoding HGF is located on chromosome 7, and consists of 18 exons and 17 introns [7]. HGF is the exclusive ligand of c-MET (cellular mesenchymal–epithelial transition factor), a membrane-bound receptor with kinase activity [8,9]. *Met*- or *Hgf*-null mutations in mice resulted in a lethal phenotype in utero, which was caused by the impaired development of placenta and liver [10,11].

HGF and c-MET expression can be upregulated by bFGF (basic fibroblast growth factor), oncostatin M, TNF-α (tumor necrosis factor-α), IL-1 (interleukin-1), IL-6 (interleukin-6), and several other cytokines [12] with the contribution of transcription factors HIF-1α (hypoxia-inducible factor 1α) and NF-κB (nuclear factor-κB).

**Figure 1:**
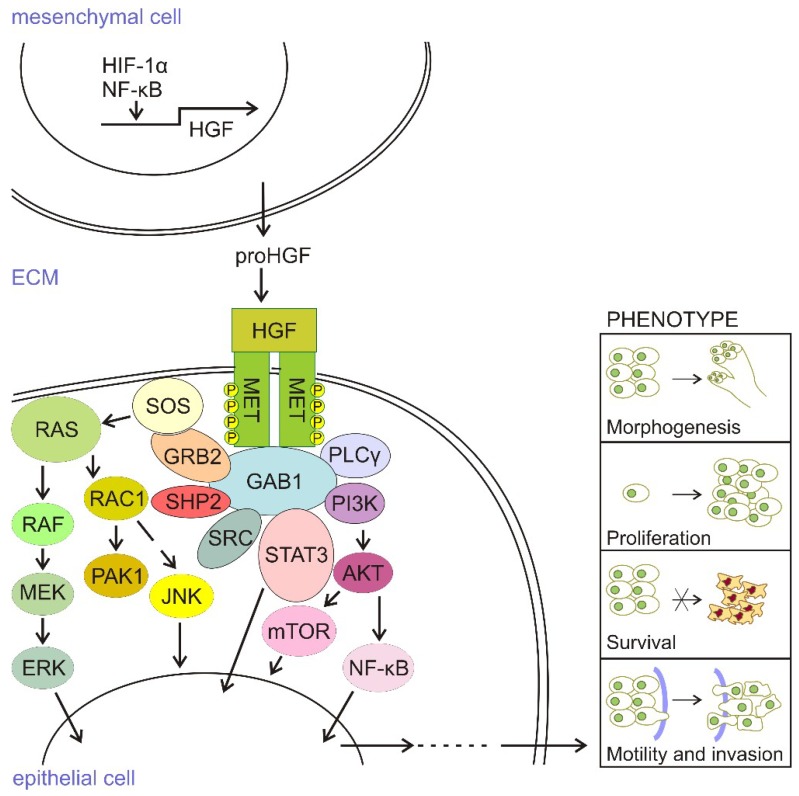
HGF/c-MET signaling. Hepatocyte growth factor (HGF) is mainly produced and secreted by mesenchymal cells as an inactive precursor, pro-HGF. Cleavage of pro-HGF to active HGF, followed by its binding to c-MET on epithelial cells, results in the dimerization of two c-MET molecules, and structural changes in a multi-substrate docking site. Recruitment of GRB2 to this site induces the autophosphorylation and binding of various transducers. Son of Sevenless (SOS) can recruit RAS-GTP to the membrane and active RAS-GTP can trigger the MAPK/ERK pathway. The autophosphorylated residue of c-MET acts also as a docking site for PI3K, which activates the AKT/mTOR pathway. Pathway activation drives transcriptional programs that mediate a plethora of cell phenotypes, including these participating in morphogenesis, proliferation, survival, motility, and invasiveness. The MET-triggered MAPK/ERK pathway is primarily involved in cell proliferation, whereas PI3K recruitment is required for the survival and induction of cell motility and invasion. RAC1-PAK signaling contributes to activities in other pathways.

HGF is secreted as a precursor, which is processed to a functional heterodimer by extracellular proteases. HGF, secreted mainly by cells of mesenchymal origin, acts in a paracrine manner on epithelial cells that express the c-MET receptor. It induces c-MET dimerization, which activates its autophosphorylation, the recruitment of various transducers such as GAB1 (Grb2-associated binder 1), GRB2 (growth factor receptor-bound protein 2), SOS (Son of Sevenless), SRC (Rous sarcoma oncogene cellular homolog), SHC (Src homology 2 domain-containing), PI3K (phosphatidylinositol 3′ kinase), PLCγ-1 (phospholipase C γ-1), SHIP2 (Src homology 2-containing inositol 5-phosphatase 1), and STAT3 (signal transducer and activator of transcription 3), among others. HGF/c-MET signaling is transmitted from the cell membrane to the nucleus mainly by the mitogen-activated protein kinase (MAPK)/extracellular signal-regulated kinase (ERK), PI3K/AKT, NF-κB, and STAT3/5 signaling pathways that influence several processes, including morphogenesis, proliferation, survival, angiogenesis, motility, and invasion (for review [13,14]). All of these processes that are triggered by HGF/c-MET signaling (Figure 1) lead to induction of the complex cellular program called “invasive growth”, which is active in diverse physiological and pathological contexts.

HGF/c-MET signaling contributes to a plethora of processes accompanying embryogenesis and tissue regeneration in adulthood, and restrains fibrosis [14,15,16,17]. The abnormal activity of the HGF/c-MET pathway has been also documented in a wide range of cancers (for review [18,19,20,21,22] and the references therein), including breast [23], esophageal [24], and cervical [25] cancers, and hepatocellular carcinoma (HCC) [26,27], brain tumors [28], head and neck [29,30], and non-small cell lung cancers (NSCLC) [31], among others.

HGF-c-MET interaction, participating in the epithelial–mesenchymal interaction, is an example of paracrine signaling as normal cells expressing c-MET do not release HGF under physiological conditions. This may change, however, after malignant transformation, as shown for several cancers [16,32,33,34,35], including melanoma [36]. This review focuses on the role of HGF/c-MET signaling in melanoma with some introductory information on its function in skin and melanocytes.

## 2. HGF/c-MET Signaling in the Skin

The skin, one of the largest organs of the human body, acts as a physical barrier between the organism and its environment. It plays an important photoprotective function that is executed by epidermal melanocytes producing melanin, which is then transferred from melanosomes, the specialized intracellular organelles, to overlaying keratinocytes, to induce pigmentation. Several autocrine and paracrine factors regulate the activity of melanocytes and other cellular components of skin, leading to dermal tissue homeostasis, and the HGF/c-MET pathway substantially contributes to this regulation.

Fibroblast-derived HGF was early recognized as a modulator of epithelial cell motility [37]. It is one of the most effective mitogenic factors secreted by fibroblasts [38]. HGF produced by dermal fibroblasts is an important mitogenic factor for keratinocytes. The first indirect evidence on the presence of c-MET on keratinocytes has been provided by showing the mitogenic and motogenic effects of HGF in these cells [39,40,41,42]. It has been shown that HGF expression and secretion can be induced in skin fibroblasts by other growth factors, EGF (epidermal growth factor), PDGF (platelet-derived growth factor), and bFGF [43]. Production of HGF in skin fibroblasts in vitro could be also stimulated by various molecules, including okadaic acid and calyculin A [44], staurosporine [45], interleukin-1 alone [46], or in combination with interferon-gamma [47] and the antibiotic polymyxin B [48]. Recently, it has been demonstrated that HGF is elevated in aged skin dermis in vivo, which is the consequence of reduced fibroblast size, and which may contribute to the age-related loss of collagen [49].

HGF is diffusely distributed in normal skin [50], and its receptor c-MET has been detected in the epidermis and dermis [51]. In the hair follicle, c-MET has been identified on keratinocytes and HGF, as released by neighboring fibroblasts in the dermal papillae [52]. HGF/c-MET signaling is involved in hair follicle morphogenesis and cycling in skin [52]. c-MET has been also identified in arrector pili muscles and blood vessels [51].

The presence of functioning c-MET on keratinocytes is crucial for wound healing in the skin. It contributes to the generation of the hyperproliferative epithelium in skin wounds [51,53,54,55]. It has been demonstrated that HGF can accelerate wound healing by promoting the dedifferentiation of epidermal cells [56], whereas the inactivation of HGF in mice with anti-HGF IgG leads to retarded cutaneous wound healing, which is associated with decreased neovascularization, and the formation of tissue granulation [51]. Recently, it has been demonstrated that HGF/c-MET signaling regulates the motogenic function of keratinocytes during skin wound healing by inducing the expression of small G protein ARF6 (ADP-ribosylation factor 6) in those cells [57].

The role of HGF/c-MET signaling in dermal tissue homeostasis can be also executed by other components of the skin [58]. In the model of tight-skin mice, which exhibits fibrosis, HGF diminished the thickening of subcutaneous dermal tissue, which was accompanied by the lowered expression of IL-4 and TGF-β [59]. HGF was demonstrated to inhibit the production of IL-4 in CD4+ T cells, as stimulated by allogenic dendritic cells. Skin dendritic cells, including Langerhans cells express c-MET, which when activated induces emigration of Langerhans cells from skin [60,61]. Thus, a similar program to MET-driven EMT (epithelial–mesenchymal transition) is mediated in Langerhans cells upon inflammatory activation, indicating an important role of HGF/c-MET signaling in skin immunity [58].

Exposure of the skin to ultraviolet (UV) radiation induces melanogenesis and the p53-dependent DNA damage response pathway [62,63,64]. HGF can inhibit UV-induced apoptosis in keratinocytes through the stimulation of the PI3K/AKT pathway [65]. Using genetically engineered mouse models, it was shown that the skin of mice overexpressing HGF and exposed to chronic suberythermal UV radiation exerted significantly enhanced sensitivity to the development of nonmelanocytic over melanocytic skin malignancies [66]. The differential expression of HGF has been induced in fibroblasts and in keratinocytes by ultraviolet A and B radiation [67,68].

## 3. HGF/c-MET Signaling in Melanocytes

HGF is not secreted by normal melanocytes, but as melanocytes express c-MET, they are receptive to HGF [40,50]. An early study has shown that HGF, as a mitogen, stimulates proliferation and melanocyte growth [39,69], and since then it is used in the culturing of melanocytes [39]. HGF received by melanocytes maintained high level of tyrosinase activity and melanin content [70]. Overexpression of HGF in transgenic mice resulted in the hyperproliferation of melanocytes in ectopic sites, melanosis in the central nervous system, and hyperpigmentation of the skin [71]. Melanocytes are cells derived from the precursor cells called melanoblasts, which originate from the neural crest cells. Studies performed with neural crest cells indicated that HGF is among the stimuli that affect melanoblast viability, proliferation, and differentiation into melanocytes [72]. Deficiency in the c-MET receptor did not lead to abnormalities in the early development of melanocytes. Using mouse embryos with a targeted *Met* null mutation (*Met*–/–), it has been demonstrated that HGF/c-MET signaling influenced, but was not required for the initial development of neural crest-derived melanocytes in vivo and in vitro [72]. However, adding HGF to neural crest cultures resulted in an elevated number of melanoblasts with increased survival potential and their differentiation into melanocytes [72].

It has been also demonstrated that HGF protects melanocytes from apoptosis [73] although it is not effective against apoptosis induced by TRAIL (TNF-related apoptosis-inducing ligand) [74]. The mechanisms behind the protective activity of HGF include upregulation of c-MET expression by MITF-M, a transcription factor of melanocytes and melanoma cells. MITF plays an important pro-survival role in melanocytes and melanoma [75]. It binds to the *MET* promoter in response to α-melanocyte stimulating hormone (α-MSH), which is markedly increased by UV radiation, or in response to forskolin, another c-AMP-elevating agent [73].

It has been shown that HGF/c-MET signaling is suppressed in melanocytes by Plexin B1, the receptor for Semaphorin D [76]. Plexin B1 can associate with c-MET, forming an oligomeric receptor–receptor complex, which inhibits the HGF/c-MET pathway by blocking SHP2 activity, followed by the abrogation of MAPK/ERK and PI3K/AKT activation in melanocytes. Interestingly, mutations in *SHP2*, blocking its activity, have been identified in LEOPARD syndrome, a disorder characterized by multiple lentigines [77].

## 4. HGF/c-MET Signaling in Melanoma

Already in the early 1990s, HGF has been shown to contribute to melanoma growth [40,70], and c-MET has been detected on melanoma cells [50,78]. The expression pattern of c-MET has been recognized as being prognostically relevant for primary cutaneous melanoma [79]. The amplification of *MET*, and the genomic rearrangements of *MET*, resulting in in-frame MET kinase fusions and c-MET activation, have been identified in melanoma [80,81].

It has been demonstrated in preclinical studies that the enhanced activity of HGF/c-MET can activate the proliferation of melanoma cells [82], increase their invasive capacity [78,83,84,85], and protect melanoma cells from apoptosis [73].

**Figure 2:**
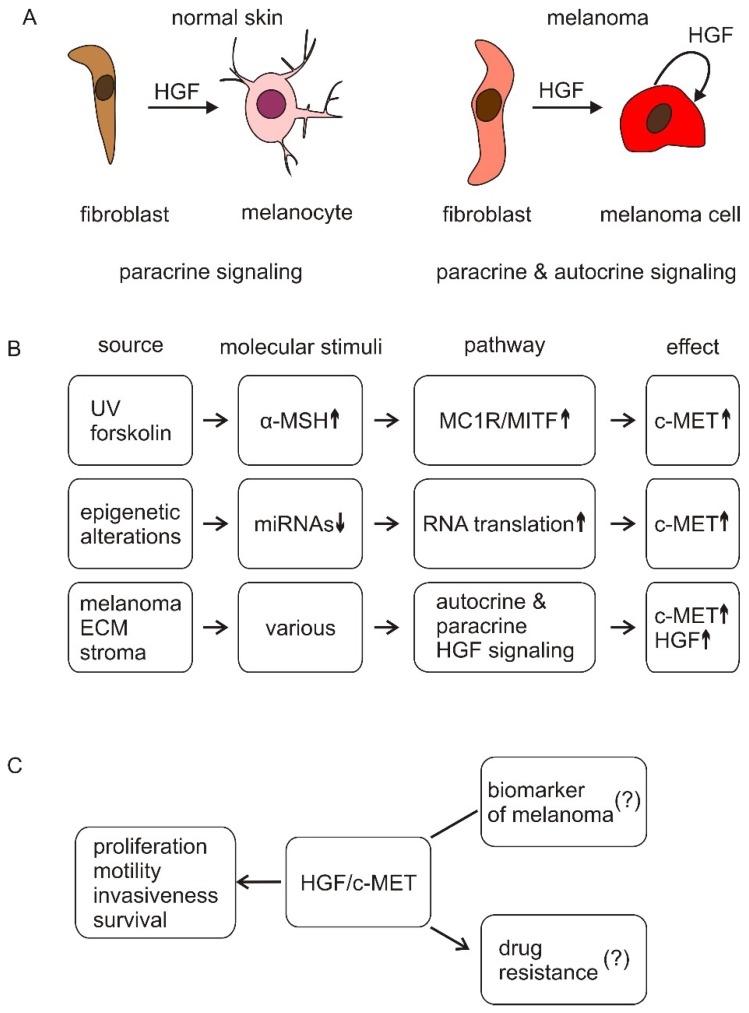
Simplified schematic illustration of the regulation of c-MET expression and activity in melanoma cells. (**A**) In normal skin, HGF is released mainly by fibroblasts to induce changes in melanocytes (paracrine signaling). In melanoma, autocrine signaling is also observed; (**B**) c-MET expression can be increased in melanoma cells through different mechanisms, including transcriptional regulation by MITF and reduced level of miRNAs targeting c-MET transcript. Activity of c-MET can be enhanced by elevated level of HGF produced and released by stromal cells (paracrine signaling) and melanoma cells (autocrine signaling) in response to diverse molecular stimuli, and with the regulatory contribution of ECM; Up- and down-arrows show enhanced and reduced levels of indicated molecules, respectively; (**C**) The enhanced activity of HGF/c-MET signaling in melanoma cells plays an important role in melanoma progression by supporting proliferation, survival, motility, and invasiveness, including niche formation. It is unclear whether HGF and/or c-MET are melanoma biomarkers, or whether the HGF/c-MET signaling contributes to the development of resistance to oncoprotein-targeted therapies.

Dissimilar to melanocytes, melanoma cells not only express c-MET, but also HGF [83]. It has been demonstrated that c-Met can be constitutively active in melanoma cells without exogenous HGF stimulation [86]. Thus, melanoma cell proliferation, survival, and acquisition of the metastatic phenotype can be supported by autocrine HGF/c-MET signaling (Figure 2).

The mechanism of HGF/c-MET signaling contribution to melanoma cell protection from apoptosis is, to some extent, similar as in melanocytes. It has been shown that cAMP-elevating agents such as α-MSH and forskolin increase the c-MET level by involving MITF-dependent transcriptional mechanism [73]. MITF silencing reduced *MET* expression in those cell lines that expressed both MITF and MET [73]. However, *MET* expression is not controlled exclusively by MITF, as no tight correlations were observed between MITF level and *MET* expression in other melanoma cell lines. Overexpression of HGF and c-MET in melanoma cells enhances cell protection from cell death, which was shown to be mediated by the activation of the MAPK/ERK and PI3K/AKT pathways [87,88], frequently modified in melanomas. Apoptosis induced by the knockdown of BRAF^V600E^ in melanoma cells can be prevented by growth factors, including HGF [89]. HGF/c-MET-dependent activation of the MAPK/ERK cascade can be decreased by the membrane receptor NOTCH and intracellular protein SPROUTY [90,91], and c-MET can contribute to the upregulation of both proteins, thus inducing negative regulators of their own activity. This interaction, however, needs to be further explored. The PI3K/AKT pathway can be activated indirectly by RAS, and/or directly by MET. This pathway can suppress apoptosis through the inactivation of proapoptotic BAD and the activation of MDM2, leading to the degradation of pro-apoptotic p53. Although the HGF/c-MET signaling also triggers NF-κB signaling, it did not contribute to the anti-apoptotic function of HGF/c-MET signaling [73]. Interestingly, it has been shown for head and neck squamous cell carcinoma that a high level of HGF can inhibit apoptosis induced by anoikis [92]. This may also be important during the dissemination of melanoma cells at distant metastases, especially when cells enter the bloodstream. Another study has reported that c-MET can promote cell survival also in a HGF-independent manner, by interacting and sequestering FAS [93].

HGF has been recognized as contributing to the regulation of the interaction between melanoma cells and their microenvironment. Autocrine expression and the activation of HGF downregulates the expression of E-cadherin and Desmoglein 1, which decouples melanoma cells from keratinocytes [94]. HGF, through the activation of the MAPK/ERK pathway, induces the expression of fibronectin and its extracellular assembly on the surface of melanoma cells, which enhances their metastatic potential [95]. HGF can be bound by surface adhesion molecule CD44, which facilitates its presentation to c-MET, and HGF binding to c-MET upregulates the expression of the CD44v6 isoform in melanoma cells [96]. It has been recently shown that HGF can contribute to the induction of the invadopodia formation, which was correlated with an enhanced invasive potential of melanoma cells [97]. HGF/c-MET signaling promotes the metastatic dissemination of melanoma cells, also through exosomes that are used for intercellular communication. These small vesicles are employed by melanoma cells to spread their proteins and nucleic acids, including microRNAs (miRNAs) [98]. It has been shown that the c-MET oncoprotein can be transported via melanoma-derived exosomes [99]. The level of activated c-MET is enhanced in this way in the bone marrow-derived cells (BMDCs). This stimulates the proangiogenic phenotype of BMDCs and their mobilization to the lungs and lymph nodes, where BMDCs facilitate the formation of a metastatic niche for melanoma cells. It has been shown that exosomes derived from Met-high B16-F10 melanoma cells containing a higher level of Met than those from Met-low cells [100]. The number of lung metastases of Met-low cells could be increased by the pretreatment of mice, with exosomes taken from Met-high melanoma cells.

HGF, c-MET and soluble MET (s-MET) are considered as biomarkers for melanoma, and they may provide a way to evaluate the response to therapy. High c-MET expression in melanoma samples has also been correlated with a poor clinical outcome [79]. Much lower levels of s-MET were detected in healthy donors and metastasis-free uveal melanoma patients, than in those with metastatic disease [101]. A high level of HGF was found in advanced disease, and in patients with progressive disease [102], and high baseline serum levels of HGF, above the median, have been correlated with lower progression-free survival (PFS) and overall survival (OS). It has been earlier shown that melanoma patients with a high stromal HGF level had a poorer response to treatment with vemurafenib, an inhibitor of BRAF^V600E^, used alone or in combination with trametinib, an inhibitor of MEK1/2, than those lacking HGF [103].

The HGF/c-MET pathway is controlled at multiple levels, and the regulation of *MET* expression by miRNAs is one of them. There are several examples supporting the notion that even slight alterations in miRNA expression levels may induce the development of pathologies, including melanoma [104]. The c-MET transcript is a target of several miRNAs, including the miR-34 family [105], miR-137 [106], and miR-144 [107]. As their expression is downregulated in melanoma, mainly by epigenetic silencing, the c-MET level is increased, leading to the enhancement of HGF/c-MET signaling.

## 5. HGF/c-MET Signaling in Melanoma that Is Resistant to Targeted Therapies

Despite the recent developments in oncoprotein-targeted drugs, melanoma is still considered a deadly disease. As a mutation in *BRAF* is present in the majority of melanomas, which leads to enhanced activity of the RAF-MEK-ERK pathway, BRAF^V600E/K^ has been chosen as the main target for the development of new therapies. There are two FDA-approved drugs targeting BRAF^V600E/K^, vemurafenib (PLX4032) and dabrafenib, but their efficacy against melanoma is not fully satisfactory, even if they are used in combination with inhibitors of the downstream kinases MEK1/2, trametinib, and cobimetinib [108]. Melanoma patients either show innate resistance to these targeted drugs, or acquired resistance is developed in the majority of patients after few months of treatment [109]. Several mechanisms of innate and acquired resistance have been identified [110,111]. As melanoma is strongly dependent on the microenvironment (TME, tumor microenvironment) [112,113], the role of the microenvironment in developing resistance has been also in focus. The role of HGF/c-MET signaling in response of melanoma to targeted therapies, and subsequent development of resistance has been investigated, which has resulted in several publications [103,114,115,116,117,118,119]. The results of these studies are not fully consistent, and they largely depend on methodology used, but the main reason for the discrepancies between results may lie in the heterogeneity of melanoma leading to the broad spectrum of activities of different pathways within one tumor. Bearing heterogeneity in mind, the results showing the role of HGF/c-MET signaling in response to targeted therapies and resistance development are grouped based on methodology, which was applied, namely as the results obtained in preclinical studies and the findings derived from clinical studies.

## 6. Preclinical Study Results

To address the potential involvement of c-MET activation in resistance to targeted therapies, a panel of 27 patient-derived melanoma cell lines were genetically characterized [115]. One cell line, LM38, exerting innate resistance to vemurafenib, showed increased activation of c-MET, which was associated with the amplification of *MET*. Vemurafenib, in combination with an inhibitor of c-MET, SU-11274, inhibited LM38 cell growth, as shown by G1 cell arrest and release of adenylate kinase in the absence of activation of caspase 3. A combination of vemurafenib and SU-11274 inhibited HGF-mediated invasion into Matrigel, preventing wound closure and inhibiting the expression of integrin β_1_. Based on these results, the authors suggested that HGF/c-MET signaling plays an important role in innate resistance to vemurafenib.

To investigate the influence of the microenvironment on melanoma response to targeted therapeutics, melanoma cells were cocultured with 18 different stromal cell lines, and among seven cell lines that conferred resistance to PLX-4720, a vemurafenib analogue, six were fibroblast cell lines [103]. The proliferation of melanoma cells cocultured with fibroblasts, and exposed to PLX-4720 was less affected by the drug than the proliferation of melanoma cells cultured alone. This was considered as an indication that stromal component(s) could rescue melanoma cells from PLX-4720-induced cell death. To find out if this rescue effect was mediated by direct fibroblast–melanoma cell contact, or by secreted factor(s), melanoma cells exposed to the drug were cultured in fibroblast-conditioned media. The results indicated that it was a secreted factor, which was identified as HGF by an antibody array-based analysis of 567 factors. The proliferation of melanoma cells was less inhibited by PLX-4720 in the medium from HGF-secreting fibroblast cultures than in the fresh medium. Next, melanoma cells were exposed to either PLX-4720 or PD-184352 (MEK1/2 inhibitor) in the presence of increasing concentrations (6.25–50 ng/mL) of recombinant HGF, HGF-neutralizing antibodies, or crizotinib, a MET inhibitor. Treatment with crizotinib reduced the HGF-mediated increase in proliferation, suggesting that rescue was MET-dependent. Relative proliferation was less reduced by drugs in the presence of HGF, whereas adding antibodies neutralizing HGF reversed this effect [103]. Authors suggested that HGF mediated innate resistance to inhibitors of BRAF^V600E/K^.

Similar conclusions were drawn from experiments performed with vemurafenib (PLX-4032) [114,120]. The acute resistance to BRAF inhibitor induced by HGF was quantified by calculating the IC_50_ (half-maximum inhibitory concentration) of vemurafenib in the presence and absence of HGF in the culture medium [114]. Among 18 melanoma cell lines analyzed, four displayed complete HGF-induced rescue, six partial and for eight melanoma cell lines addition of HGF did not affect melanoma cell response to vemurafenib. Among 12 additional BRAF^V600E^ melanoma cell lines, HGF markedly antagonized vemurafenib sensitivity only in five lines [114]. As shown in this study, the correlation between the expression and the HGF rescue effect in melanoma cells treated with vemurafenib was not high (*r*^2^ = 0.56). In the study of Lito et al., HGF attenuated the sensitivity to vemurafenib in five out of eight BRAF^V600E^ melanoma cell lines [120]. These results indicate that HGF can suppress effects of BRAF^V600E^ inhibition but only in selected melanoma cell lines.

In a more recently published report [118], it has been demonstrated that the proliferation and phenotype of six BRAF^V600E^ patient-derived melanoma cells were not detectably influenced by exogenous HGF. Cell distribution in the cell cycle, the expression of *CCND1*, the activity of signaling pathways crucial for melanoma, including the RAF/MEK/ERK pathway, WNT/β-catenin pathway, and NF-κB signaling were very similar when different growth factors, HGF, EGF and bFGF, were used in the culture medium alone, in combination or were omitted. This suggests that growth factors, including HGF, are not necessary for BRAF^V600E^ melanoma cell growth in vitro. Moreover, the effects of vemurafenib or trametinib on melanoma cells were also very similar in all of these growth conditions [118]. Interestingly, the presence of growth factors did not influence the percentages of subpopulations of Ki-67^high^ (proliferating) cells and CD271 (NGFR)^high^ (stem-like, crest-like) cells, and these percentages were reduced similarly by drugs in the presence or absence of different growth factors. Altogether, these results suggest that growth factors, including HGF, do not substantially alter the effects of acute treatment of melanoma cells with inhibitors of the MAPK/ERK pathway, vemurafenib and trametinib.

The half-life of exogenous HGF protein is short, and high levels of HGF cannot be kept in vivo, even by repeated infusions of HGF [53]. Results of in vivo studies on a possible role of HGF/c-MET signaling in the development of resistance to BRAF^V600E^ inhibitors were not conclusive, which might be partially due to sample sizes.

## 7. Clinical Results

HGF was detected in melanoma-associated stromal cells in 68% BRAF^V600E^ patient-derived biopsies from patients prior to treatment (23/34 biopsies) [103], whereas, when 10 on-treatment biopsies were analyzed, HGF expression was found to be enhanced in 50% of them, compared to its pre-treatment level. In another study, the ability of stromal cells, which are few in number and located at the periphery of metastatic nodules, to counteract treatment via HGF was questioned [117]. In this study, stromal or parenchymal HGF immunoreactivity has been evaluated as a biomarker for melanoma response to RAF inhibitors, and the results were not unambiguous. In another study, the evaluation of patients did not provide satisfactory results to validate HGF as a biomarker for melanoma response to targeted therapies [102]. Also, the MET clinical score was not a statistically significant predictive factor for therapy with vemurafenib [116].

There is no reliable method to quantify the HGF level directly in a living patient [117]. The levels of growth factors, which are secreted from multiple sources, dynamically change. In patients enrolled onto the BRIM2 clinical trial, pretreatment plasma HGF levels ranged from 0.033 to 7.2 ng/mL, with a median value of 0.33 ng/mL [114]. Assays for the reliable detection of HGF and MET levels are still under development [121,122].

## 8. Targeting HGF/c-MET Signaling in Melanoma

The inhibitors of HGF/c-MET signaling are developed to target mainly c-MET (Table 1), but their efficacy is largely limited by resistance. Cabozantinib showed clinical activity in patients with metastatic melanoma, including uveal melanoma [123], although it is unclear whether this is exclusively due to the inhibitory effects on c-MET, as cabozantinib also inhibits VEGF receptors and the AXL receptor tyrosine kinase. The inhibition of c-Met activity alone by Crizotinib was sufficient to strongly inhibit the metastasis of uveal melanoma in a mouse model [124]. A Phase II trial of adjuvant crizotinib in high-risk uveal melanoma following definitive therapy (NCT02223819) is ongoing. SU11274 was shown to induce the apoptosis and differentiation of melanoma cells in vitro [125]. The antitumor activity of SU11274 was also observed in a melanoma xenograft model [86,126]. SU11274, used for intraperitoneal administration achieved a significant inhibitory effect on liver metastasis induced by the intrasplenic injection of metastatic melanoma cells [86]. On the contrary, SU11274 was shown to increase the tumorigenicity and alter the bioenergetic state of melanoma cells [127]. Interestingly, it has been demonstrated that combination of SU11274 and vemurafenib inhibited the growth of melanoma cells with constitutively activated c-MET more efficiently than SU11274 alone [115]. Foretinib inhibited the migration and invasion capacity of B16F10 melanoma cells, with an IC_50_ of 21 nM [128]. PHA-665752, another c-MET inhibitor, inhibited the migration of NRAS-mutated melanoma cells toward HGF [129]. PHA-665752, while suppressing the phosphorylation of c-MET, significantly inhibited the PI3K/AKT pathway activity and motility of uveal melanoma cells in vitro, and tumor growth in nude mice [130]. Tivantinib (ARQ 197) is clinically tested as a c-MET inhibitor [131]. Preliminary evidence of the therapeutic potential of combinatorial therapy, tivantinib, and sorafenib, was obtained in adult melanoma patients [132]. The synergistic effect of vemurafenib and tivantinib on cell viability was observed in c-MET-expressing melanoma cells that harbored a mutation leading to BRAF^V600E^ [126]. In vitro, tivantinib did not inhibit the autophosphorylation of c-MET, and its cytotoxic activity was independent of its ability to bind c-MET [133]. The dietary flavonoid quercetin, whose safety for clinical application was reviewed [134], has been shown to inhibit melanoma cell migration and invasion in an in vitro study, and to prevent melanoma lung metastasis in vivo [135]. This antimetastatic activity of quercetin might be partially due to the inhibition of HGF/c-MET signaling by the suppression of c-MET dimerization, its phosphorylation, and as a consequence, the attenuation of downstream transducers [36]. A therapeutic development strategy for quercetin as an antimelanoma agent has been presented [136]. Recently, it has been demonstrated that quercetin can inhibit the phosphorylation of JAK2 and STAT1 in interferon gamma-primed keratinocytes [137].

## 9. Conclusions

HGF is an important mediator of interactions between cancer cells and stroma cells [140]. Moreover, several elements of the microenvironment, including ECM components [19] can either promote or inhibit HGF/c-MET signaling. Thus, the discrepancies between the results obtained in preclinical studies, and in clinics, it might be a consequence of the differences in the microenvironment used in the experimental settings or present in the patients’ malignancies.

The contribution of the HGF/c-MET signaling to wound healing, including skin repair, inspires the development of drugs with agonistic activities, as a part of regenerative medicine. From an opposite perspective, the role of the HGF/c-MET pathway in the progression of several tumors, including melanoma, stimulates research towards the development of inhibitors of this pathway. Both agonists and antagonists influencing the HGF/c-MET signaling are currently being tested in clinical trials. These “two-pronged approaches”, already early recognized [141], are being closely examined for possible applications in tissue repair and cancer treatment without inducing deleterious side-effects.

## Figures and Tables

**Table 1:** Inhibitors of HGF/c-MET signaling investigated in melanoma cells, in preclinical and clinical studies.

Name of Inhibitor	Designed/Assessed Activity	Type of Experiments	References
CabozantinibXL184 BMS-907351	Inhibitor of c-MET	Clinical trial	NCT00940225 [123,138,139]
Crizotinib PF-02341066	Adenosine triphosphate (ATP)-competitive inhibitor of catalytic activity of c-MET	Preclinical in vivo clinical trial (uveal melanoma)	[103,124] NCT02223819 (ongoing)
Foretinib EXEL-2880	ATP-competitive inhibitor of c-MET	Preclinical in vitro Preclinical in vivo	[128]
PHA-665752	Inhibitor of Y1234 and Y1235 in catalytic region of c-MET	Preclinical in vitro	[129,130]
SU11274	Selective inhibitor of Y1234 and Y1235 in catalytic region of c-MET	Preclinical in vitro Preclinical in vivo	[86,115,125,126,127]
Tivantinib ARQ 197	Non-ATP-competitive inhibitor of c-MET; binding to dephosphorylated c-MET	Preclinical in vitro Preclinical in vivo Clinical trial	[126,131,132,133] NCT00827177
E7050	ATP-competitive inhibitor of c-MET	Clinical trial	NCT01433991
Quercetin	Inhibitor of c-MET phosphorylation and dimerization	Preclinical in vitro Preclinical in vivo	[36,135]

https://clinicaltrials. gov.
